# Membrane Vesicles Derived From *Clostridium botulinum* and Related Clostridial Species Induce Innate Immune Responses *via* MyD88/TRIF Signaling *in vitro*

**DOI:** 10.3389/fmicb.2022.720308

**Published:** 2022-02-03

**Authors:** Nobuhide Kobayashi, Kimihiro Abe, Sachiyo Akagi, Mayu Kitamura, Yoshitake Shiraishi, Aki Yamaguchi, Masahiro Yutani, Sho Amatsu, Takuhiro Matsumura, Nobuhiko Nomura, Noriyuki Ozaki, Nozomu Obana, Yukako Fujinaga

**Affiliations:** ^1^Department of Bacteriology, Graduate School of Medical Sciences, Kanazawa University, Kanazawa, Japan; ^2^Faculty of Life and Environmental Sciences, University of Tsukuba, Ibaraki, Japan; ^3^Department of Functional Anatomy, Graduate School of Medical Sciences, Kanazawa University, Kanazawa, Japan; ^4^Department of Forensic Medicine and Pathology, Graduate School of Medical Sciences, Kanazawa, Japan; ^5^Microbiology Research Center for Sustainability, University of Tsukuba, Ibaraki, Japan; ^6^Transborder Medical Research Center, Faculty of Medicine, University of Tsukuba, Ibaraki, Japan

**Keywords:** *Clostridium botulinum*, *Clostridium sporogenes*, *Clostridium scindens*, membrane vesicles, innate immunity, toll-like receptors, MyD88, TRIF

## Abstract

*Clostridium botulinum* produces botulinum neurotoxin complexes that cause botulism. Previous studies elucidated the molecular pathogenesis of botulinum neurotoxin complexes; however, it currently remains unclear whether other components of the bacterium affect host cells. Recent studies provided insights into the role of bacterial membrane vesicles (MVs) produced by some bacterial species in host immunity and pathology. We herein examined and compared the cellular effects of MVs isolated from four strains of *C. botulinum* with those of closely related *Clostridium sporogenes* and two strains of the symbiont *Clostridium scindens*. MVs derived from all strains induced inflammatory cytokine expression in intestinal epithelial and macrophage cell lines. Cytokine expression was dependent on myeloid differentiation primary response (MyD) 88 and TIR-domain-containing adapter-inducing interferon-β (TRIF), essential adaptors for toll-like receptors (TLRs), and TLR1/2/4. The inhibition of actin polymerization impeded the uptake of MVs in RAW264.7 cells, however, did not reduce the induction of cytokine expression. On the other hand, the inhibition of dynamin or phosphatidylinositol-3 kinase (PI3K) suppressed the induction of cytokine expression by MVs, suggesting the importance of these factors downstream of TLR signaling. MVs also induced expression of Reg3 family antimicrobial peptides *via* MyD88/TRIF signaling in primary cultured mouse small intestinal epithelial cells (IECs). The present results indicate that MVs from *C. botulinum* and related clostridial species induce host innate immune responses.

## Introduction

*Clostridium botulinum* is an anaerobic, Gram-positive, spore-forming bacteria that produces botulinum neurotoxin (BoNT) and causes the neurotoxic disease botulism. BoNT cleaves soluble NSF attachment protein receptor proteins at presynaptic terminals and impedes acetylcholine secretion, which leads to systemic nerve paralysis and lethal respiratory failure (Pirazzini et al., 2017). BoNT forms progenitor toxin complexes (PTCs) with non-toxic components that are absorbed by the intestines in the case of food poisoning (Fujinaga and Popoff, 2018). *Clostridium botulinum* serotype A1 strains produce three types of PTC: M-PTC, L-PTC, and LL-PTC (a dimer of L-PTC). L-PTC contains BoNT, non-toxic non-HA (NTNHA), and HA, whereas M-PTC consists of only BoNT and NTNHA (Gu and Jin, 2013). Serotype A1 L-PTC enters the host body from intestinal microfold (M) cells *via* interactions with glycoprotein 2 (GP2) on M cell surfaces and hemagglutinin (HA), one of the non-toxic components of L-PTC (Matsumura et al., 2015; Kobayashi et al., 2019). Botulinum HA also interacts with E-cadherin and disrupts epithelial barrier function, which increases toxin invasion (Sugawara et al., 2010, 2014; Lee et al., 2014). M-PTC also causes botulism; however, the mechanisms by which M-PTC passes through the intestinal barrier remain unclear.

Naturally occurring human botulism is classified into three disease types based on the intoxication route: foodborne, intestinal, and wound botulism. Foodborne botulism is food poisoning caused by the ingestion of toxin-containing foods (Rasetti-Escargueil et al., 2020). Foodborne botulism may occur when the toxin is present in food even if *C. botulinum* is not alive. On the other hand, colonization of the host intestines by *C. botulinum* causes infant botulism and adult intestinal toxemia botulism (collectively termed intestinal botulism; Rasetti-Escargueil et al., 2020). In this case, ingested botulinum spores from food or the environment germinate in the intestines and subsequently produce BoNT. Healthy adults are resistant to botulinum infections, whereas infants are susceptible because of the immaturity of their intestinal microbiota (Shirey et al., 2015). Some adults with risk factors, such as previous gastrointestinal surgery, anatomical bowel abnormalities, inflammatory bowel disease, and antimicrobial treatment, are also susceptible to intestinal botulism (Harris et al., 2020). It is important to note that patients with intestinal botulism are chronically exposed to *C. botulinum*; however, it is unknown whether components other than the BoNT complex affect its pathogenesis. In addition, limited information is currently available on host immune responses to *C. botulinum*.

Recent studies provided insights into the roles of membrane vesicles (MVs) secreted by bacteria in host–microbe interactions and pathogenesis (Cecil et al., 2019). MVs are nanosized vesicles composed of a lipid bilayer and are produced by most Gram-negative and -positive bacteria (Toyofuku et al., 2019). They contain various molecules, such as proteins, DNA, RNA, plasmids, and virulence factors, which may modulate host metabolism and immune responses (Cecil et al., 2019; Toyofuku et al., 2019). MVs induce innate immune responses to host cells by delivering pathogen-associated molecular patterns (PAMPs), such as lipopolysaccharide (LPS) and peptidoglycan *via* pattern recognition receptors (PRRs; Irving et al., 2014; Vanaja et al., 2016). In the lungs, MVs derived from commensal *Bacteroides* and *Prevotella* promote pulmonary fibrosis *via* interleukin (IL)-17B-mediated immune responses (Yang et al., 2019). Pathogenic *Escherichia coli* deliver their virulence factors to host cells *via* MVs (Rueter and Bielaszewska, 2020). *Vibrio cholerae* uses MVs as a decoy for host antimicrobial factors to increase adaptation and colonization fitness *in vivo* (Zingl et al., 2020). MVs also exert beneficial effects on host homeostasis. *Bacteroides fragilis*-derived MVs suppress experimental colitis and inflammation by inducing IL-10 production from dendritic cells *via* TLR2, which promotes regulatory T-cell responses (Shen et al., 2012). MVs derived from *Akkermansia muciniphila* were previously shown to upregulate tight junction molecules in colonic epithelial cells and improve intestinal barrier function (Chelakkot et al., 2018). Therefore, MVs from various bacteria regulate host homeostasis and pathogenesis.

In the present study, we focused on the cellular effects of MVs derived from *C. botulinum* and related clostridial species. We obtained MVs from four strains of *C. botulinum* and compared them with those of closely related *Clostridium sporogenes* and two strains of symbiont *C. scindens*. MVs derived from all of these bacteria induced inflammatory responses in both intestinal epithelial and macrophage cell lines. Cytokine expression was dependent on myeloid differentiation primary response (MyD) 88 and TIR-domain-containing adapter-inducing interferon-β (TRIF), essential adaptors for toll-like receptors, and TLR1/2/4, which are pattern recognition receptors for bacterial cell surface components. The inhibition of dynamin-dependent endocytosis or phosphatidylinositol-3 kinase (PI3K) decreased cytokine induction by clostridial MVs, whereas the inhibition of actin polymerization, which was crucial for MV uptake, was dispensable. MVs also induced the expression of Reg3 family antimicrobial peptides (AMPs) *via* MyD88/TRIF pathway in primary cultured mouse intestinal epithelial cells (IECs). The present results demonstrated that MVs secreted by clostridia, including pathogenic and commensal, induced innate immune responses in host cells *via* MyD88/TRIF signaling.

## Materials and Methods

### Bacterial Strains and Isolation of MVs

MVs were isolated from the culture supernatants of *C. botulinum* type A strain 62A, 7I03-H, type B strain Okra, and type E strain Iwanai (Kozaki et al., 1974, 1995; Inoue et al., 1996; Yokosawa et al., 2009). *Clostridium sporogenes* strain ATCC7955 and *C. scindens* strain ATCC35704 were obtained from ATCC and *C. scindens* strain VPI12708 (JCM10418) from RIKEN BRC. Spores or glycerol stock was inoculated into 5 ml of cooked meat medium (0.1 g/ml cooked meat, 0.3% glucose, and 0.3% soluble starch; for *C. botulinum* and *C. sporogenes*) or BHIS-F medium (Marion et al., 2019; for *C. scindens*) at 30°C (*C. botulinum* type E Iwanai) or 37°C (other strains) under anaerobic conditions using AnaeroPack Kenki (Mitsubishi Gas Chemical) for 1–2 days until overgrowth. Four milliliters of the culture was then transferred into 400 ml of toxin-producing medium (1% polypeptone, 1% lactalbumin hydrolysate, 0.5% peptone, 0.5% yeast extract, 1% glucose, 0.2% NaCl, and 0.05% cysteine-HCl, pH 7.8; Yokosawa et al., 2009) and incubated under the conditions described above for 4–5 days. The isolation of clostridial MVs from culture supernatants was previously reported (Obana et al., 2017). Briefly, the culture was centrifuged at 6,000 × *g* at 4°C for 30 min. The supernatant was filtrated with a 0.45-μm PES filter and subsequently ultracentrifuged at 150,000 × *g* at 4°C for 1 h. MV pellets were resuspended and purified by density-gradient centrifugation using OptiPrep (Abbott Diagnostics Technologies AS) at 100,000 × *g* at 4°C for 3 h. Bands containing MVs were transferred and pelleted by ultracentrifugation at 150,000 × *g* at 4°C for 1 h. Purified MV pellets were resuspended in cell suspension (CS) buffer (10 mM HEPES-KOH and 0.85% NaCl, pH 6.8). The protein concentration of MVs was quantified using the Pierce BCA Protein Assay Kit (Thermo Fisher Scientific) in the presence of 2% sodium dodecyl sulfate (SDS) to eliminate the effects of lipids in MVs.

### Transmission Electron Microscopy

Purified MVs were allowed to adhere to Thin Carbon film-coated TEM grids (ALLIANCE Biosystems) for 30 s and were negatively stained with EM Stainer (Nisshin EM). Stained MVs were analyzed using an H-7650 transmission electron microscope (Hitachi).

### Nanoparticle Tracking Assay

Size distribution analysis was performed on a NanoSight NS600 (Malvern Panalytical) according to the manufacturer’s instructions. Purified MVs were injected into a sample chamber of the NanoSight system. Brownian motion of the MVs in solution was visualized and captured by a microscope camera. The acquired movie data were analyzed using NTA software (version 3.2.16; Malvern Panalytical).

### Coomassie Brilliant Blue Staining

Samples were mixed with 2× or 5× SDS sample buffer and incubated at 95°C for 5 min. In CBB staining, proteins were separated on 12.5% polyacrylamide gels and stained using the standard CBB staining protocol.

### Cell Culture, MV Stimulation, Chemical Inhibition, and Conditioned Medium Production

RAW264.7, CMT-93, and Caco-2 cells were propagated in Dulbecco’s modified Eagle’s medium (DMEM, Nacalai Tesque) containing 10% (v/v) fetal bovine serum (FBS), 100 U/ml penicillin, and 100 μg/ml streptomycin. In the MV stimulation assay, cells were seeded on 48-well plates. Cells were then treated with 10 (for RAW264.7) or 50 (for CMT-93 and Caco-2) μg of MVs for 6 h. In the chemical inhibition assay, cells were pre-treated with 5 μM CuCPT22, 1 μM TAK242, 20 μM cytochalasin D, 80 μM dynasore, 20 μg/ml filipin III, or 10 μM LY294002 for 1–2 h. Medium was then replaced with fresh medium containing both MVs and an inhibitor. Cells were incubated for 1 (uptake assay) or 6 h (RT-qPCR).

L-WRN cells (purchased from ATCC) were propagated in DMEM containing 10% FBS, 100 U/ml penicillin, 100 μg/ml streptomycin, 0.5 mg/ml G-418, and 0.5 mg/ml hygromycin B. Production of L-WRN conditioned medium was performed by a previously described protocol with simple modification (Miyoshi and Stappenbeck, 2013). Briefly, cells were cultured in culture medium (without G-418 and hygromycin) until becoming over-confluent. Then, cells were washed and add basal medium [Advanced DMEM/F-12 (Invitrogen) containing 20% FBS, 1% GlutaMAX (Gibco), 10 mM HEPES (pH7.5), 100 U/ml penicillin, and 100 μg/ml streptomycin]. After 24 h incubation, the medium was removed to a tube and fresh medium was added. The conditioned medium was centrifuged at 2,000 × *g* for 5 min, and the supernatant was transferred to a bottle. The bottle was stored at 4°C. Every 24 h, conditioned medium was collected to the same bottle. After the 10th to 12th collection, pooled conditioned medium was dispensed and stored at −80°C until use. Thawed conditioned medium was diluted by half with basal medium (50% L-WRN conditioned medium).

### Mice

MyD88/TRIF double knockout (dKO) mice (C57BL/6 background) were obtained from the Oriental Bio Service and maintained under specific pathogen-free environment. Wild-type (WT) C57BL/6J mice were purchased from Japan SLC. 4-week-old female mice were used for experiments. All animal studies were performed according to approved protocols by the Kanazawa University for euthanasia prior to harvesting tissues.

### Isolation of Mouse Bone Marrow-Derived Macrophages

The limbs of WT or MyD88/TRIF dKO mice were collected. The skin and muscle were peeled off, and the bones were soaked into 70% ethanol. The bone marrow cells in the bones were pushed out into RPMI1640 medium (Nacalai Tesque) containing 2% FBS. The collected cells (1.25 × 10^5^ cells per a well in 48-well plate) were cultured in complete RPMI-1640 medium [RPMI-1640 containing 10% FBS, 100 U/ml penicillin, 100 μg/ml streptomycin, 55 μM mercaptoethanol, 1% Glutamax, and 12.5 mM HEPES (pH7.5)], with macrophage colony-stimulating factor (M-CSF, 20 ng/ml, PeproTech) for 6 days. The medium was changed on day 3. After 6 days culture, cells were stimulated with 10 μg/ml MVs for 6 h.

### Primary Culture of Mouse Small Intestinal Epithelial Cells

The duodenum was removed from WT or MyD88/TRIF dKO mice and cut into 5-mm segments. The segments were incubated in cold 2 mM EDTA in PBS for 5 min and washed by pipetting. The segments were incubated in 2 mM cold EDTA in PBS for 30 min, and crypts were isolated by pipetting with cold HBSS. Dissociated crypts were through 70-μm cell strainers. The crypts were resuspended in advanced DMEM/F12 medium; then, the number of crypts were counted, and the crypts were resuspended in 50% L-WRN conditioned medium supplemented with 10 μM Y-27632. Next, the crypts were directly plated in a 96-well plate and medium was changed on day 3 (without Y-27632). At day 6, cells were stimulated with 50 μg/ml MVs for 24 h.

### Cytotoxicity Assay

The cytotoxicity of MVs was assessed using the cell count reagent SF (Nacalai Tesque) according to the manufacturer’s instructions. This assay is based on the cellular reduction of WST-8 by mitochondrial dehydrogenases and reflects cell viability as well as correlating with cell numbers. Briefly, RAW264.7 and Caco-2 cells were cultured in 96-well plates at cell densities of 1,000 and 5,000 cells/well, respectively. After 24 h, cells were treated with 10 (RAW264.7) or 50 (Caco-2) μg/ml MVs for 6 or 24 h, and 10 μl/well of the cell count reagent SF was then added. After 1 h, the cellular reduction of WST-8 was measured as absorbance at 450 nm. Values were reported as the percentage of WST-8 reduction relative to untreated cells.

### Real-Time Quantitative PCR

Total RNA was prepared from cells using TRIzol (Thermo Fisher Scientific). First-strand cDNA synthesis was completed using ReverTra Ace qPCR RT Master Mix (TOYOBO). Quantitative PCR reactions were conducted in QuantStudio 3 (Thermo Fisher Scientific) using KOD SYBR qPCR Mix (TOYOBO). The specific primers used are shown in Supplementary Table 1.

### Enzyme-Linked Immunosorbent Assay

RAW264.7 cells were cultured in 24-well plate with 500 μl medium. Cells were then treated with 10 μg/ml MVs for 30 h. Culture supernatant was removed and centrifuged at 2,000 *g* for 10 min. The supernatant was diluted with 10% FBS/PBS. Cytokine concentration was quantified using ELISA kits, OptEIA Mouse IL-6 ELISA Set, OptEIA Mouse TNF ELISA Set (both from BD Biosciences), and Mouse IL-1 beta SimpleStep ELISA Kit (Abcam), according to manufacturer’s protocol.

### Fluorescent Labeling of MVs

MV labeling with fluorescein isothiocyanate (FITC) was performed according to a previously reported protocol with a simple modification (Kesty et al., 2004). Briefly, purified MVs were pelleted by ultracentrifugation. MV pellets were resuspended in 100 μg/ml FITC isomer-I/0.1 M carbonate buffer (pH 9.0) and incubated at 4°C o/n. FITC-labeled MVs were washed with CS buffer 3 times and resuspended in CS buffer.

### MV Uptake Assay

RAW264.7, CMT-93, and Caco-2 cells were plated on a cover glass placed in a 24-well plate. In the case of CMT-93 and Caco-2, cells were starved in serum-free medium for 2 h before the addition of MVs. Cells were then treated with 50 or 100 μg/ml FITC-labeled MVs and incubated at 37°C for 1 h. After the incubation, cells were washed with PBS three times. Regarding immunofluorescence, cells were fixed in 3.7% (w/v) formaldehyde in PBS for 15 min and permeabilized with 0.1% (v/v) Triton X-100 in 1% (w/v) BSA-containing PBS for 15 min. Cells were then treated with Alexa Fluor 568-conjugated phalloidin (Thermo Fisher Scientific) and Hoechst 33342 (DOJINDO). After washing with PBS, cells were observed/photographed using the laser scan confocal microscope IX71 (PLYMPUS). To quantify internalized MVs, cells were lysed with passive lysis buffer (Promega). Hoechst 33342 (final 1/2,000) was added to the cell lysate, and fluorescence intensity was measured with the plate reader Spark (Tecan).

### Statistical Analysis

Differences between mean values for multiple groups were analyzed by a one-way ANOVA with Tukey’s multiple comparisons test or a two-way ANOVA with Šídák’s multiple comparisons test using Prism9 (GraphPad Software).

## Results

### MVs Secreted From *Clostridium botulinum* and Related Clostridia

**Figure 1:**
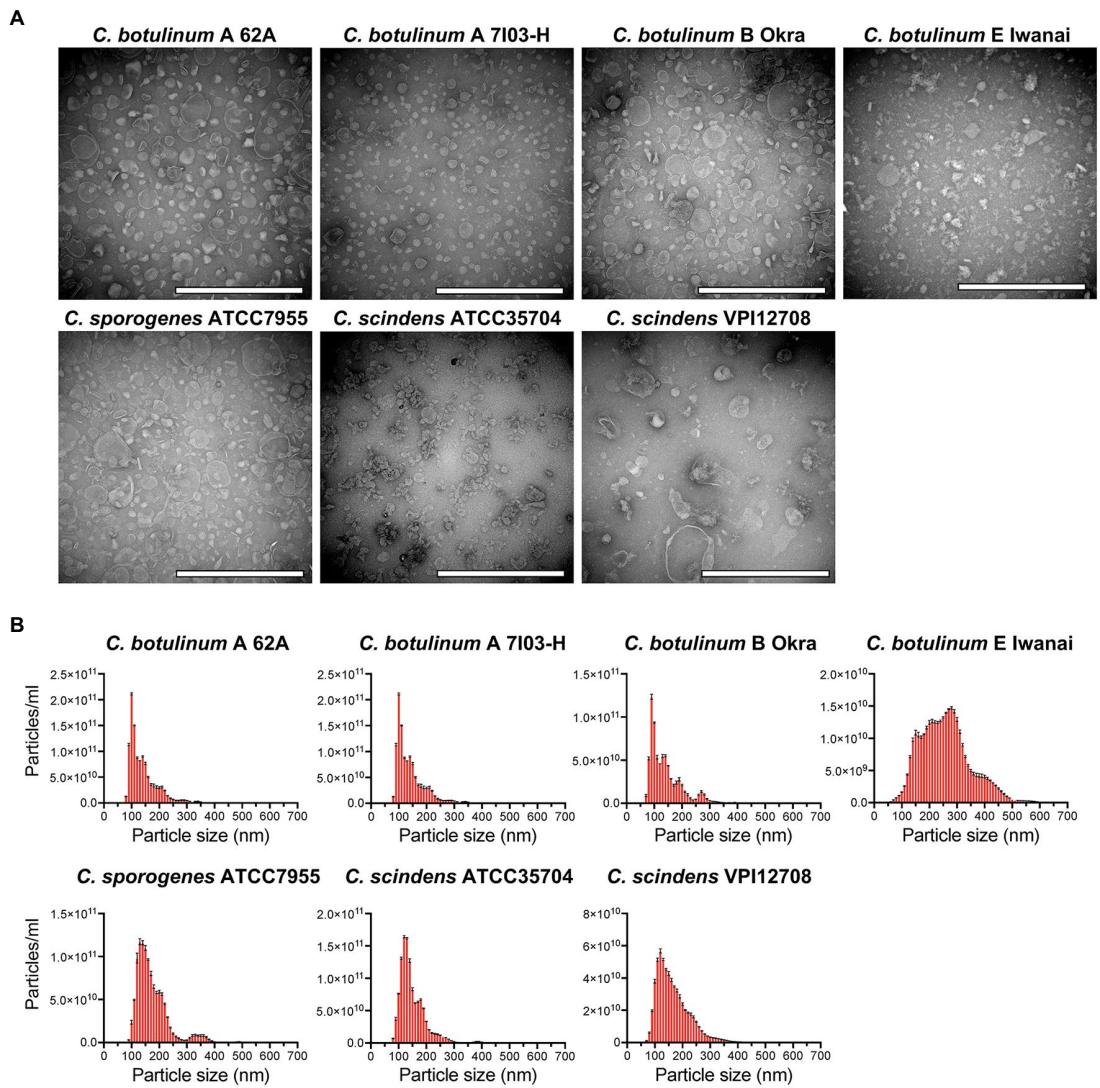
Isolation of membrane vesicles (MVs) from *Clostridium botulinum*, *Clostridium sporogenes*, and *Clostridium scindens*. **(A)** Transmission electron microscopy (TEM) analysis of purified MVs. Bars, 200 nm. **(B)** Size distributions of MVs analyzed by nanoparticle tracking assay (NTA). Error bars indicate the SD of three measurements. Data are representative of at least two lots of MVs purified on different date.

To clarify whether *C. botulinum* secretes MVs under *in vitro* culture conditions, we purified MVs from culture supernatants by ultracentrifugation and gradient density centrifugation. *Clostridium botulinum* is defined by its ability to produce BoNT and is classified into four groups based on its bacteriological characteristics (Collins and East, 1998). We obtained MVs from three strains (type A 62A, type A 7I03-H, and type B Okra) of group I *C. botulinum* and one strain (type E Iwanai) of group II (Carter and Peck, 2015). We purified MVs from *C. sporogenes* ATCC7955, which is closely related to group I *C. botulinum*, for comparison (Carter and Peck, 2015). We also obtained MVs from two strains of *C. scindens*, ATCC35704 and VPI12708, as beneficial commensal clostridia (Buffie et al., 2015). TEM analysis revealed that all strains produced spherical MVs (Figure 1A). Among them, a lot of irregular-shaped structures were observed in *C. botulinum* type E Iwanai MVs, which could not be removed by gradient density centrifugation. We also evaluated particle size of MVs by NTA (Figure 1B). In most strains, the particle size peaked at 100–150 nm and was mainly distributed in the 50–400 nm range. Particle size of MVs from *C. botulinum* type E Iwanai showed irregular pattern compared to other strains, which may be due to irregular-shaped structures observed in TEM inspection. The CBB staining patterns of MVs differed from that of the whole cell lysate, suggesting that MVs have a distinctive protein composition (Supplementary Figure 1).

### Clostridial MVs Induce Inflammatory Cytokine Expression in Macrophage and Intestinal Epithelial Cell Lines

**Figure 2:**
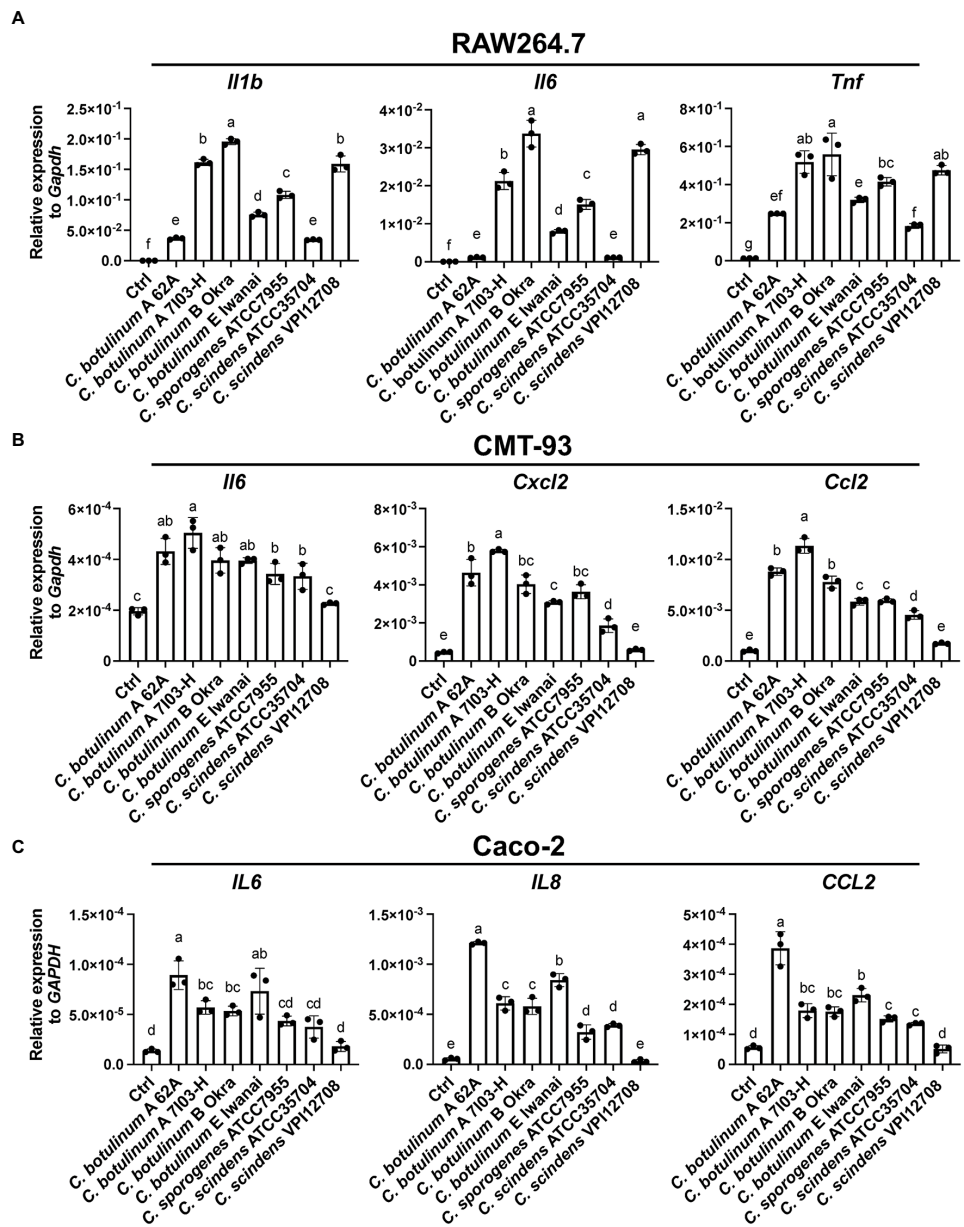
Real-time quantitative PCR (RT-qPCR) analysis of cytokine gene induction by clostridial MVs in **(A)** RAW264.7, **(B)** CMT-93, and **(C)** Caco-2 cells. Cells were treated with 10 **(A)** or 50 **(B,C)** μg/ml MVs for 6 h. Ctrl, control. Means that do not share a letter are significantly different (*p* < 0.05). Error bars indicate SD. *n* = 3, a one-way ANOVA with Tukey’s multiple comparisons *post hoc* test. Data are representative of two independent experiments.

MVs derived from *C. perfringens* induce inflammatory cytokine expression in J774A.1 macrophage-like cell lines, suggesting that other clostridial MVs also activate inflammatory responses (Obana et al., 2017). *Clostridium botulinum* and related clostridial MVs were normalized by protein concentrations, and gene expression was analyzed by real-time quantitative PCR (RT-qPCR). MVs from all strains induced inflammatory cytokine expression in the mouse macrophage-like cell line, RAW264.7 (Figure 2A). *Clostridium botulinum* type B Okra MVs induced the highest expression levels of the cytokines *Il1b*, *Il6*, and *Tnf*, while those from *C. botulinum* type A 62A and *C. scindens* ATCC35704 induced the lowest levels. IL-1β is produced as cytosolic precursor pro-IL-1β, its secretion needs cleavage by Caspase1 *via* activation of inflammasomes. Thus, we treated RAW264.7 cells with MVs from four strains (*C. botulinum* type A 62A, A 7I03-H, E Iwanai, and *C. sporogenes* ATCC7955) and evaluated secretion of cytokine secretion in culture supernatant by ELISA (Supplementary Figure 2). MVs from *C. botulinum* type A 62A, A 7I03-H, and *C. sporogenes* ATCC7955 induced IL-1β, IL-6, and Tnf secretion at different levels. *Clostridium botulinum* type E Iwanai MVs induced Tnf and very weak IL-6 secretion, but failed to induce secretion of IL-1β. Because epithelial cells are the frontline barrier against gut microbiota and enteric pathogens, we next analyzed inflammatory responses against MVs in intestinal epithelial cell lines by RT-qPCR. In the mouse colonic epithelial cell line CMT-93, *C. botulinum* type A 7I03-H MVs induced the highest expression levels of *Il6*, *Cxcl2*, and *Ccl2*, while *C. scindens* VPI12708 MVs induced the lowest (Figure 2B). In Caco-2, a cell line of human colorectal cancer, *C. botulinum* type A 62A MVs induced the highest expression levels of *IL6*, *IL8*, and *CCL2*, while *C. scindens* VPI12708 MVs induced the lowest (Figure 2C). These results demonstrated that clostridial MVs induce inflammatory responses in mammalian cells, with an induction capacity that is dependent on the host cell type rather than the bacterial strain.

**Figure 3:**
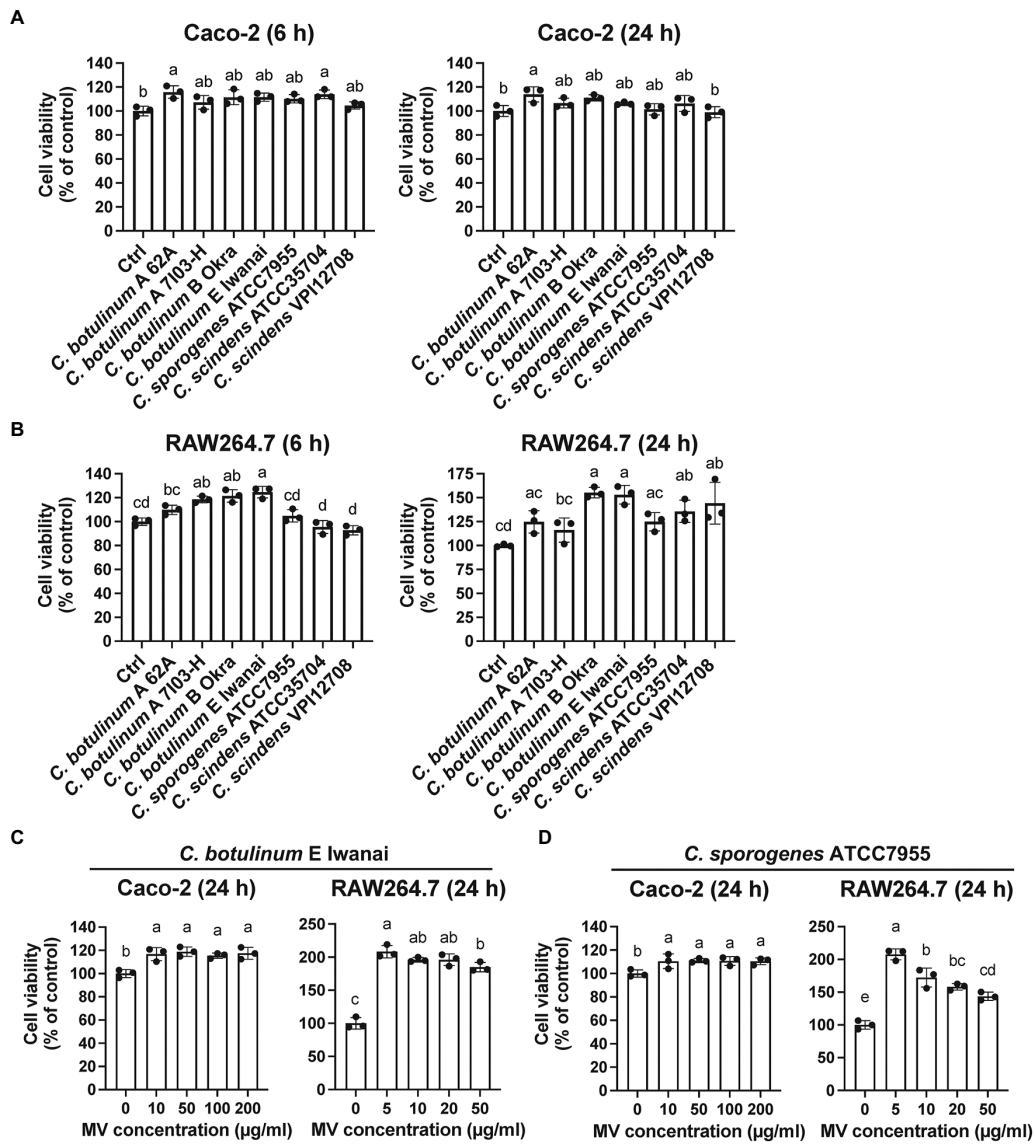
Cytotoxicity of clostridial MVs in **(A)** Caco-2 and **(B)** RAW264.7 cells. Cells were treated with 10 or 50 μg/ml MVs for 6 or 24 h. Cell viability was evaluated by measuring intracellular dehydratase activity. **(C)** Cells were treated with the different doses of MVs (0–200 μg/ml for Caco-2 and 0–50 μg/ml for RAW264.7) derived from *C. botulinum* type E Iwanai and **(D)** *C. sporogenes* ATCC7955 for 24 h. Error bars indicate SD. Means that do not share a letter are significantly different (*p* < 0.05). *n* = 3, a one-way ANOVA with Tukey’s multiple comparisons *post hoc* test. Data are representative of two independent experiments.

MVs from some pathogenic bacteria exhibit cytotoxicity to host cells (Rivera et al., 2010; Kunsmann et al., 2015; Nicholas et al., 2017). To investigate whether clostridial MVs are cytotoxic, we examined the viability of cells treated with MVs. MVs from all strains were not cytotoxic in Caco-2 or RAW264.7 cells after 6 or 24 h of treatment at a single concentration (Figures 3A,B). We subsequently treated cells with MVs from *C. botulinum* type E Iwanai and *C. sporogenes* ATCC7955 at several concentrations for 24 h, but no cytotoxicity was observed even at high concentrations (Figures 3C,D). These observations suggest that clostridial MVs have minimal cytotoxicity against host cells under our experimental conditions.

### Innate Immune Responses Against Clostridial MVs Are Mediated by MyD88/TRIF Signaling

**Figure 4:**
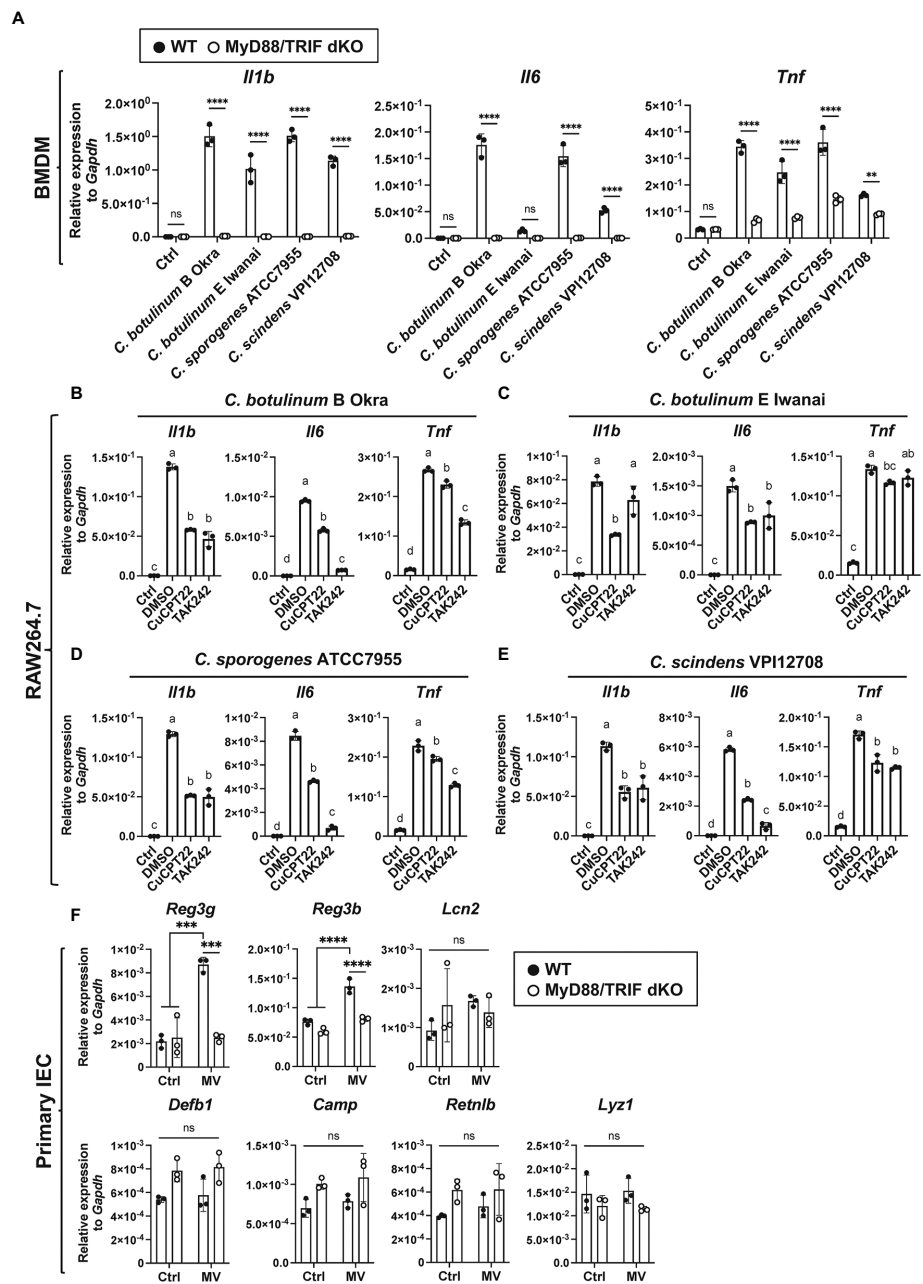
MyD88/TRIF-dependent innate immune responses against clostridial MVs. **(A)** WT or MyD88/TRIF dKO BMDMs were treated with 10 μg/ml MVs derived from *C. botulinum* type E Iwanai, *C. sporogenes* ATCC7955, or *C. scindens* VPI12708 for 6 h. Gene expression levels were measured using a RT-qPCR analysis. Ctrl, control; **, *p* < 0.01; ****, *p* < 0.0001; ns, not significant. Error bars indicate SD. *n* = 3, a two-way ANOVA with Šídák’s multiple comparisons *post hoc* test. **(B–E)** Effects of TLR inhibitors on cytokine expression induced by MVs. Cells were pre-treated with 5 μM CuCPT22, 1 μM TAK242, which inhibits TLR1/2, and TLR4, respectively, for 1.5 h. Cells were then treated with 10 μg/ml MVs derived from **(B)** *C. botulinum* type B Okra, **(C)** *C. botulinum* type E Iwanai, **(D)** *C. sporogenes* ATCC7955, or **(E)** *C. scindens* VPI12708, with inhibitors for 6 h. Ctrl, control. Means that do not share a letter are significantly different (*p* < 0.05). Error bars indicate SD. *n* = 3, a one-way ANOVA with Tukey’s multiple comparisons *post hoc* test. **(F)** AMP expression in primary cultured mouse small intestinal epithelial cells (IECs) treated with 50 μg/ml *C. botulinum* type E Iwanai MVs for 24 h. Ctrl; control. ***, *p* < 0.001; ****, *p* < 0.0001; ns, not significant. Error bars indicate SD. *n* = 3, a two-way ANOVA with Šídák’s multiple comparisons *post hoc* test. Data are representative of two independent experiments.

MVs induce inflammatory responses mediated by PRRs, such as TLR2 (Obana et al., 2017). To identify the PRRs responsible for inflammatory responses against clostridial MVs, we subsequently analyzed the contribution of TLRs to cytokine induction using bone marrow-derived macrophages (BMDMs) derived from MyD88/TRIF dKO mice. MyD88 and TRIF are intracellular adaptor proteins required for overall TLR signaling (Yamamoto et al., 2003). Cytokine induction by MVs from four strains (*C. botulinum* type B Okra, type E Iwanai, *C. sporogenes* ATCC7955, and *C. scindens* VPI12708) in MyD88/TRIF dKO BMDMs was prominently lower than that of WT BMDMs, indicating that TLR signaling is critical for inflammatory responses against MVs (Figure 4A). We next analyzed the contribution of specific TLRs using chemical inhibitors. We focused on TLR2 and TLR4 to identify TLRs which recognize bacterial cell surface components contained in MVs. TLR2 forms heterodimer with TLR1 or TLR6 and recognizes bacterial components, such as peptidoglycan from Gram-positive bacteria (Takeuchi and Akira, 2001). TLR4 recognizes both Gram-negative and Gram-positive bacterial components, such as LPS and lipoteichoic acid, respectively. CuCPT22 binds TLR1/2 and competes ligand binding (Cheng et al., 2012). TAK242 inhibits interaction between TLR4 and intracellular adaptor proteins, Toll/interleukin-1 receptor domain-containing adaptor protein (TIRAP) or Toll/interleukin-1 receptor domain-containing adaptor protein inducing interferon-β-related adaptor molecule (TRAM; Matsunaga et al., 2011). RAW264.7 cells were pre-treated with CuCPT22 and TAK242. These cells were then incubated with MVs in the presence of inhibitors, and cytokine expression was analyzed by RT-qPCR. In our experimental condition, CuCPT22 affects TLR4 signaling partially (Supplementary Figures 3B,C). The induction of *Il1b* and *Il6* expression by *C. botulinum* type B Okra MVs was suppressed by both inhibitors, whereas the induction of *Tnf* expression was moderately decreased (Figure 4B). Similar effects were observed when cells were treated with MVs from *C. sporogenes* ATCC7955 and *C. scindens* VPI12708 (Figures 4D,E). When cells were treated with *C. botulinum* type E Iwanai MVs, CuCPT22 significantly suppressed expression of *Il1b*, *Il6*, and *Tnf*, but TAK242 only suppressed *Il6* (Figure 4C). We also found the attenuation of TLR1/2 and TLR4 suppressed cytokine expression by *C. botulinum* type E Iwanai MVs in CMT-93 cells (data not shown). Notably, the cytokine induction was lower, but still observed in MyD88/TRIF dKO BMDMs, suggesting that PRRs other than TLRs play roles in the detection of MVs (Supplementary Figure 3A). These results indicate that clostridial MVs stimulate PRRs *via* not only a single ligand, but also various PAMPs.

The expression of several AMPs produced by IECs is regulated by intestinal microbiota (Shin and Seeley, 2019; Blyth et al., 2020; Brooks et al., 2021). Thus, we analyzed expression levels of several AMPs in primary cultured small IECs treated with *C. botulinum* E Iwanai MVs. Among them, *Reg3g* and *Reg3b*, Reg3 family AMPs expressed in the gastrointestinal tract, was strongly induced by MV stimulation dependent on MyD88/TRIF signaling pathway (Figure 4F).

### Clostridial MVs Are Taken Up by Actin Filament Dependent Pathway in RAW264.7 Cells

**Figure 5:**
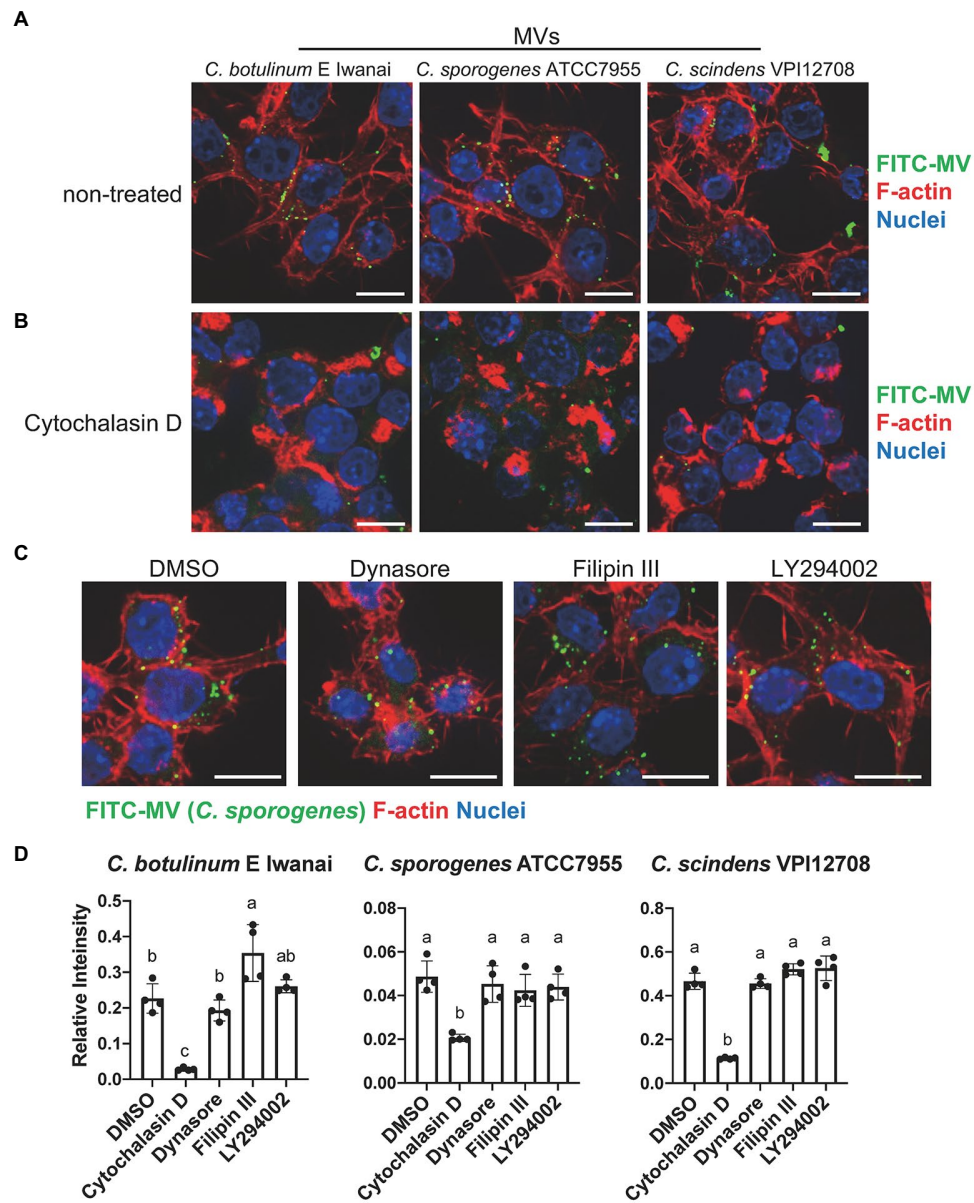
Uptake of clostridial MVs in RAW264.7 macrophages. **(A)** A fluorescence microscopy analysis of MV localization in RAW264.7 cells. Cells were treated with 50 μg/ml FITC-labeled MVs for 1 h. Bars, 10 μm. **(B)** Inhibitory effects of 10 μM cytochalasin D on MV uptake. **(C)** Effects of 80 μM dynasore, 20 μg/ml filipin III, and 10 μM LY294002, which inhibit dynamin, lipid rafts, and PI3K, respectively, on MV uptake. Bars, 10 μm. **(D)** Quantification of fluorescence intensity of cell lysates. FITC intensity was normalized by that of Hoechst33342. Means that do not share a letter are significantly different (*p* < 0.05). Error bars indicate SD. *n* = 4, a one-way ANOVA with Tukey’s multiple comparisons *post hoc* test. Data are representative of two independent experiments.

We investigated whether clostridial MVs are taken up by mammalian cells. To visualize their localization in cells, MVs from *C. botulinum* type E Iwanai, *C. sporogenes* ATCC7955, and *C. scindens* VPI12708 were labeled with FITC. FITC-MVs were internalized into the cytoplasm in RAW264.7, CMT-93, and Caco-2 cells (Figure 5A; Supplementary Figure 4). To identify the entry route of MVs in RAW264.7 cells, these cells were treated with FITC-MVs in the presence of uptake inhibitors. Cells treated with cytochalasin D, which inhibits actin polymerization-dependent endocytosis pathway, such as phagocytosis and macropinocytosis, reduced cytoplasmic FITC-MVs from all three strains (Figure 5B). The other inhibitors, dynasore, filipin III, and LY294002, which suppress dynamin-dependent endocytosis, caveola-mediated endocytosis, and PI3K activity (PI3K is important for macropinocytosis), respectively, did not affect FITC-MV uptake into the cytoplasm (Figure 5C). The quantification of FITC-MV uptake based on the fluorescent intensity of cell lysates demonstrated that only cytochalasin D suppressed intracellular FITC levels (Figure 5D). These results indicate that actin polymerization-dependent pathway is a major route for clostridial MV uptake in RAW264.7 cells.

### Dynamin and PI3K Activity Are Critical for Cytokine Induction by Clostridial MVs in RAW264.7 Cells

**Figure 6:**
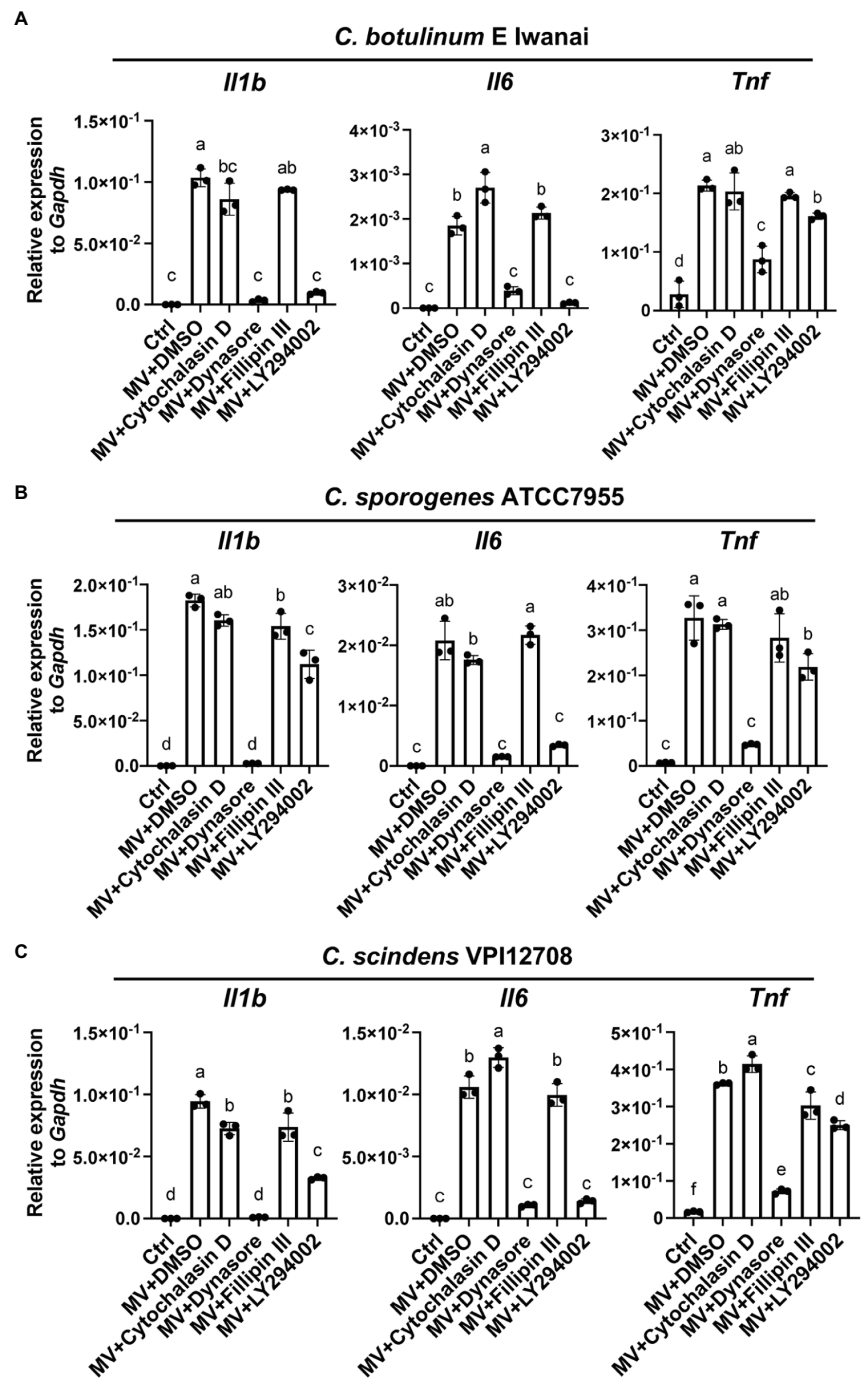
Dynamin-dependent endocytosis and PI3K activity are responsible for the induction of cytokine expression by clostridial MVs in RAW264.7 cells. Cells were pre-treated with 20 μM cytochalasin D, 80 μM dynasore, 20 μg/ml filipin III, or 10 μM LY294002 for 2 h. Cells were then treated with 10 μg/ml MVs derived from **(A)** *C. botulinum* type E Iwanai, **(B)** *C. sporogenes* ATCC7955, or **(C)** *C. scindens* VPI12708, with inhibitors for 6 h. Gene expression levels were measured using a RT-qPCR analysis. Ctrl, control. Means that do not share a letter are significantly different (*p* < 0.05). Error bars indicate SD. *n* = 3, a one-way ANOVA with Tukey’s multiple comparisons *post hoc* test. Data are representative of two independent experiments.

To clarify whether the inhibition of MV uptake affects the induction of cytokine expression, we examined gene expression levels in RAW264.7 cells treated with MVs and the inhibitors described above. Dynasore strongly inhibited the induction of *Il1b* and *Il6* expression by MVs from *C. botulinum* type E Iwanai and moderately suppressed that of *Tnf* expression (Figure 6A). LY294002 also strongly suppressed the induction of *Il1b* and *Il6* expression and slightly inhibited that of *Tnf* expression. However, cytochalasin D and filipin III did not inhibit the induction of cytokine expression. Similar effects were observed when cells were treated with MVs from *C. botulinum* type B Okra (Supplementary Figure 5). Dynasore strongly inhibited the induction of cytokine expression by MVs from *C. sporogenes* ATCC7955 and *C. scindens* VPI12708 (Figures 6B,C). LY294002 strongly suppressed the induction of *Il6* expression and moderately inhibited that of *Il1b* and *Tnf* expression. These results demonstrate that dynamin-dependent endocytosis and PI3K activity are crucial for inflammatory responses against clostridial MVs, whereas MV uptake dependent on actin polymerization is dispensable.

## Discussion

Previous studies provided molecular insights into the BoNT complex. However, the roles of other components of *C. botulinum* remain unclear. In the present study, we isolated MVs from *C. botulinum* and related clostridia and examined their effects on mammalian cell lines. Although MVs from the outer membrane of Gram-negative bacteria have been the focus of research in the last decade, limited information is currently available on Gram-positive bacterium-derived MVs, particularly clostridial MVs. To the best of our knowledge, this is the first study to report the cellular effects of MVs secreted by *C. botulinum*, *C. sporogenes*, and *C. scindens*.

**Figure 7:**
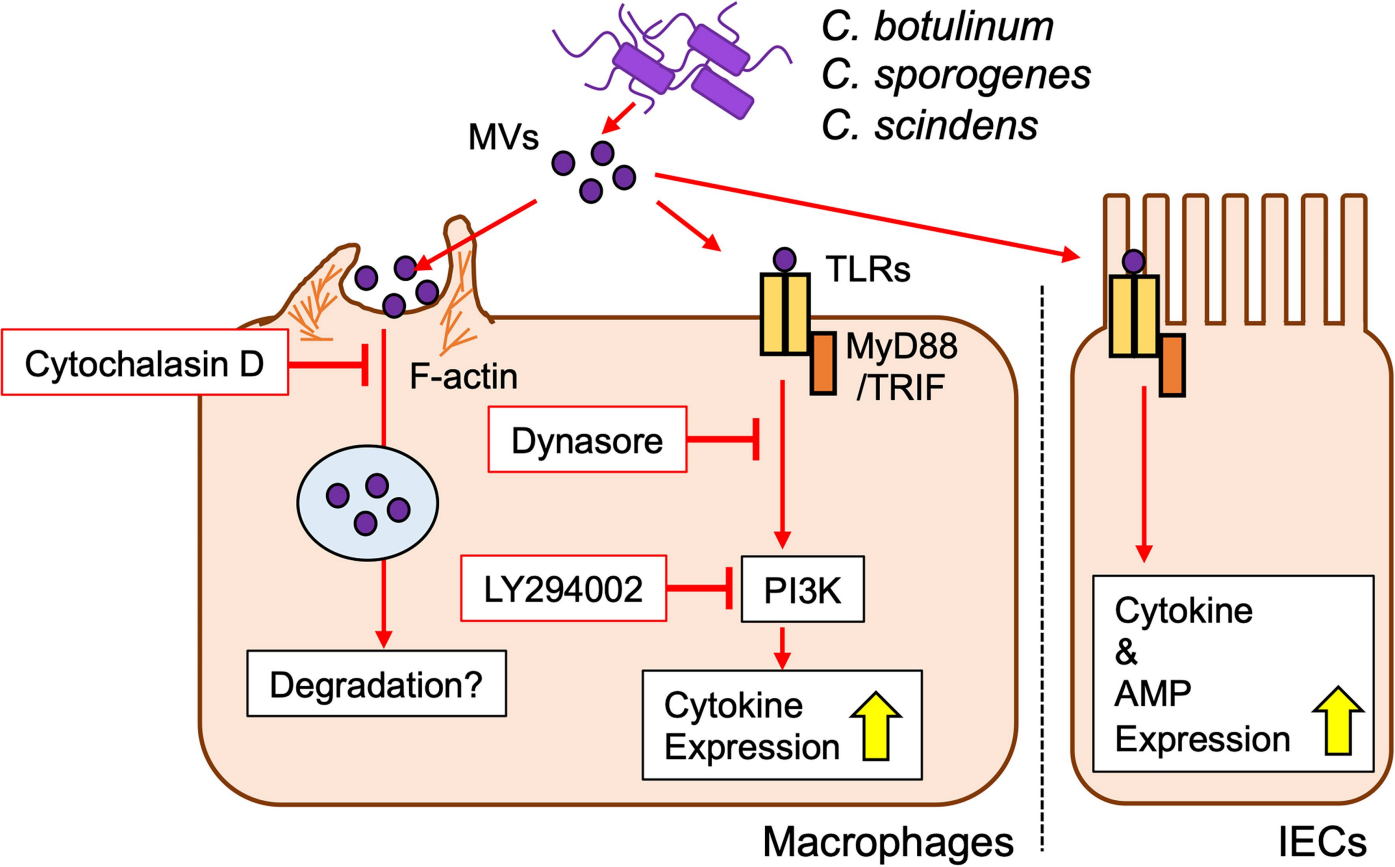
Proposed model of innate immune responses against clostridial MVs in macrophages and IECs.

MVs from all strains tested in the present study induced inflammatory cytokine expression in cell lines. The induction capacity was dependent on the host cell type rather than the bacterial strain/species. Depletion of MyD88/TRIF strongly decreased cytokine induction by clostridial MVs, indicating the importance of TLR signaling. In the case of *C. perfringens* MVs, the induction of inflammatory responses was dependent on TLR2 (Obana et al., 2017). We identified TLR4 as a receptor for MVs from *C. botulinum* type B Okra, *C. botulinum* type E Iwanai, *C. sporogenes* ATCC7955, and *C. scindens* VPI12708, in addition to TLR1/2. TLR4 is a receptor for the LPS of Gram-negative bacteria, but also recognizes the lipoteichoic acid of Gram-positive bacteria (Takeuchi and Akira, 2001). Contribution of other TLRs to clostridial MV recognition is a future issue. Notably, the cytokine inductivity of MVs was still observed in MyD88/TRIF dKO cells, suggesting that MVs were also recognized by PRRs other than TLRs. Some bacterial MVs activate nucleotide-binding oligomerization domain (NOD) 1/2, cytosolic receptors for the cell wall components, γ-D-glutamyl-meso-diaminopimelic acid, and muramyl dipeptide, respectively (Wolf and Underhill, 2018). MVs from *Helicobacter pylori*, *Pseudomonas aeruginosa*, and *Neisseria gonorrhoeae* deliver peptidoglycans to host cells and induce the activation of NOD1 (Kaparakis et al., 2010; Irving et al., 2014). *Staphylococcus aureus* MVs were shown to activate NOD2 and TLR2/7/8/9 to induce the expression of cytokines (Bitto et al., 2021). Accordingly, NOD receptors may contribute to clostridial MV recognition. In the present study, TLR1/2 and TLR4 (and other PRRs) were both important for the induction of cytokine expression by clostridial MVs, indicating that MVs contain not only a single ligand, but various PAMPs that activate several PRRs. PRR expression patterns differed in each cell line, which may contribute to the induction of different cytokine expression patterns by individual MVs (Cario et al., 2000). Thus, clostridial MVs, both pathogenic and commensal, deliver PAMPs to host cells and induce inflammatory responses *via* PRRs (Figure 7). Of note, secretion of cytokine proteins induced by MVs did not correlate gene expression levels. Although this study focused on the signaling pathway of innate immune responses against clostridial MVs, protein-level analysis is necessary to clarify the actual inflammatory response *in vivo*.

Various routes for MV entry into host cells have been reported, such as clathrin-dependent endocytosis, caveolin-mediated endocytosis, lipid rafts, and membrane fusion, depending on the bacterial strain (O’Donoghue and Krachler, 2016). However, the mechanisms by which clostridial MVs enter host cells have not yet investigated. We herein demonstrated that actin polymerization-dependent pathway was the main route for the uptake of clostridial MVs into RAW264.7 cells. Membrane ruffling triggered by reconstitution of actin cytoskeleton is essential for phagocytosis by macrophages, suggesting that MVs are taken by phagocytosis in RAW264.7 cells. MV internalization was not reduced by inhibition of dynamin-dependent endocytosis, lipid raft formation, and PI3K activity. On the other hand, the induction of cytokine expression by MVs was strongly suppressed when cells were treated with dynamin or PI3K inhibitors, whereas the inhibition of actin polymerization did not attenuate inflammatory responses. Therefore, internalized MVs may be degraded in, such as phagolysosomes, which prevents the activation of PRRs. A limitation of the present study is that the effects of labeling proteins in MVs with FITC on protein function and MV behavior remain unclear. Besides, the fluorescence intensity of FITC is sensitive to intracellular pH and diminishes in low pH organelles, such as lysosomes (Lanz et al., 1997). In addition, we only used chemical inhibitors to analyze uptake route of MVs. Dynasore is known as a dynamin inhibitor, but also impacts lipid raft formation, actin stabilization, and membrane raffling, which may affect the phenotypes (Preta et al., 2015). Further investigations are needed to clarify the detailed uptake route and subcellular localization of MVs.

TLR2/4 induces TIRAP-MyD88 signaling at the plasma membrane and then translocates to early endosomes *via* endocytosis to activate TRAM-TRIF signaling (Kagan et al., 2008; Nilsen et al., 2015). The suppression of inflammatory responses to clostridial MVs by dynamin inhibitors may be due to impeded TLR endocytosis and TRAM-TRIF signaling. PI3K-Akt signaling cross-talks with TLR signaling, which upregulates inflammatory cytokine expression in the TLR2 pathway and negatively regulates the TLR4 pathway (Fukao and Koyasu, 2003; Ruse and Knaus, 2006). PI3K-mediated inflammatory responses may reflect the dominance of TLR2 signaling in the clostridial MV stimulation.

Inflammatory responses are closely related to the pathogenesis and aggravation of many infections and diseases. Previous studies demonstrated that MVs from various bacteria induced innate immune responses, suggesting the involvement of MVs in diseases, whereas the *in vivo* role of MVs remains unknown. Pioneering work revealed that MVs from commensal bacteria exacerbated pulmonary fibrosis *via* host immune responses in mice (Yang et al., 2019). In accordance with these findings, *C. botulinum* may regulate botulism by activating inflammatory cytokine expression *via* MV secretion. Alternatively, the innate immune system originally protects hosts from pathogen infections, such as *Clostridioides difficile* (Hasegawa et al., 2011). In the present study, *C. botulinum* E Iwanai MVs induced expression of Reg3 family AMPs, *Reg3g* and *Reg3b*, *via* MyD88/TRIF signaling. Reg3γ is an AMP that kills Gram-positive bacteria by binding to peptidoglycan layer, and Reg3β exerts bactericidal activity against Gram-negative bacteria *via* direct binding to LPS (Shin and Seeley, 2019). Thus, the host immune system may recognize *C. botulinum* infection *via* MVs and eliminate it from the body. Further studies using mouse models will clarify the role of *C. botulinum* MVs in botulism and provide novel insights into their involvement in host diseases.

In conclusion, we herein demonstrated that MVs derived from *C. botulinum* and related clostridial species activated innate immune responses in host cells *via* MyD88/TRIF signaling, thereby expanding our understanding of clostridia and broadening the horizon of future MV research.

## Data Availability Statement

The original contributions presented in the study are included in the article/Supplementary Material; further inquiries can be directed to the corresponding author.

## Ethics Statement

The animal study was reviewed and approved by Kanazawa university animal experiment committee.

## Author Contributions

NK and YF: conceptualization and funding acquisition. NK, NOb, YS, NOz, SAm, and TM: methodology. NK, KA, SAk, MK, YS, AY, NOb, and YF: investigation. MY and NN: resources. NK: writing (original draft). YF: writing (review and editing). All authors contributed to the article and approved the submitted version.

## Funding

The present study was supported by grants from the Japan Society for the Promotion of Science (20K17462 to NK and 18H02654 to YF), AMED (20wm0325025h0001 to NK), Ohyama Health Foundation (NK), Takeda Science Foundation (NK), and Yakult Foundation (YF).

## Conflict of Interest

The authors declare that the research was conducted in the absence of any commercial or financial relationships that could be construed as a potential conflict of interest.

## Publisher’s Note

All claims expressed in this article are solely those of the authors and do not necessarily represent those of their affiliated organizations, or those of the publisher, the editors and the reviewers. Any product that may be evaluated in this article, or claim that may be made by its manufacturer, is not guaranteed or endorsed by the publisher.
