# Supplementary material for: Membrane Vesicles Derived From Clostridium botulinum and Related Clostridial Species Induce Innate Immune Responses via MyD88/TRIF Signaling in vitro
Source: Front Microbiol. 2022 Feb 3;13:720308. doi: 10.3389/fmicb.2022.720308 (PMC8851338; doi:10.3389/fmicb.2022.720308)
Supplement: Supplementary file 1 [file Table_1.DOCX]

**Supplementary Figure 1.**

SDS-PAGE pattern of proteins present in clostridial MVs. Gels are stained by CBB. Data are representative of at least two independent experiments.

**Supplementary Figure 2.**

Cytokine secretion by RAW264.7 cells stimulated with MVs. Cells were treated with 10 µg/mL MVs for 30 h. Protein concentration in culture supernatant was evaluated by ELISA. Error bars indicate SD. Means that do not share a letter are significantly different (P<0.05). n=3, a one-way ANOVA with Tukey’s multiple comparisons post-hoc test.

**Supplementary Figure 3.**

**(A)** BMDMs derived from MyD88/TRIF dKO mice were treated with 10 µg/mL MVs for 6 h. **(B-C)** RAW264.7. cells were pre-treated with 5 µM CuCPT22, 1 µM TAK242 for 2 h. Cells were then treated with **(B)** 10 ng/mL Pam3CSK4, a TLR2 ligand, or **(C)** 500 ng/ml MPLA, a TLR4 ligand with inhibitors for 6 h. Gene expression levels were measured using a RT-qPCR analysis. Ctrl, control. Means that do not share a letter are significantly different (*P*<0.05). Error bars indicate SD. n=3, a one-way ANOVA with Tukey’s multiple comparisons post-hoc test. Data are representative of two independent experiments.

**Supplementary Figure 4.**

Fluorescence microscopy analysis of *C. botulinum* type E Iwanai MVs taken up by CMT-93 and Caco-2 cells. Cells were treated with 100 µg/mL FITC-labeled MVs for 1 h. Bars, 10 µm. Data are representative of at least two independent experiments.

**Supplementary Figure 5.**

RAW264.7. cells were pre-treated with 20 µM cytochalasin D, 80 µM dynasore, 20 µg/ml filipin III, or 10 µM LY294002 for 2 h. Cells were then treated with 10 µg/mL MVs derived from *C. botulinum* type B Okra, with inhibitors for 6 h. Gene expression levels were measured using a RT-qPCR analysis. Ctrl, control. Means that do not share a letter are significantly different (*P*<0.05). Error bars indicate SD. n=3, a one-way ANOVA with Tukey’s multiple comparisons post-hoc test. Data are representative of two independent experiments.

**Supplementary Table 1.**

List of specific primers used in the RT-qPCR analysis.

| **Species** | **Target gene** | **Accession No.** | **Forward** | **Reverse** |
| --- | --- | --- | --- | --- |
| *mouse* | *Gapdh* | NM_001289726.1 | TGTGTCCGTCGTGGATCTGA | TTGCTGTTGAAGTCGCAGGAG |
|  | *Il1b* | NM_008361.4 | TGTGAAATGCCACCTTTTGA | GGTCAAAGGTTTGGAAGCAG |
|  | *Il6* | NM_031168.2 | TGATGCACTTGCAGAAAACA | ACCAGAGGAAATTTTCAATAGGC |
|  | *Tnf* | NM_013693.3 | AGTTCTATGGCCCAGACCCT | CTCCTCCACTTGGTGGTTTG |
|  | *Cxcl2* | NM_009140.2 | TGAACAAAGGCAAGGCTAACTG | CAGGTACGATCCAGGCTTCC |
|  | *Ccl2* | NM_011333.3 | CACTCACCTGCTGCTACTCA | GCTTGGTGACAAAAACTACAGC |
|  | *Reg3g* | NM_011260.2 | CAGACAAGATGCTTCCCCGT | GCAACTTCACCTTGCACCTG |
|  | *Reg3b* | NM_011036.1 | ACTCCCTGAAGAATATACCCTCC | CGCTATTGAGCACAGATACGAG |
|  | *S100a8* | NM_013650.2 | AAATCACCATGCCCTCTACAAG | CCCACTTTTATCACCATCGCAA |
|  | *Lcn2* | NM_008491.1 | TGGCCCTGAGTGTCATGTG | CTCTTGTAGCTCATAGATGGTGC |
|  | *Defb1* | NM_007843.3 | AGGTGTTGGCATTCTCACAAG | GCTTATCTGGTTTACAGGTTCCC |
|  | *Camp* | NM_009921.2 | GCTGTGGCGGTCACTATCAC | TGTCTAGGGACTGCTGGTTGA |
|  | *Retnlb* | NM_023881.4 | AAGCCTACACTGTGTTTCCTTTT | GCTTCCTTGATCCTTTGATCCAC |
|  | *Lyz1* | NM_013590.4 | GAGACCGAAGCACCGACTATG | CGGTTTTGACATTGTGTTCGC |
| *human* | *GAPDH* | NM_002046.7 | GGACCTGACCTGCCGTCTAG | GAGGAGTGGGTGTCGCTGTT |
|  | *IL6* | NM_000600.5 | TGGCTGAAAAAGATGGATGCT | TCTGCACAGCTCTGGCTTGT |
|  | *IL8* | NM_000584.4 | TTGGCAGCCTTCCTGATTTC | TGGTCCACTCTCAATCACTCTCA |
|  | *CCL2* | NM_002982.4 | TCGCTCAGCCAGATGCAAT | TGGCCACAATGGTCTTGAAG |
